# Underutilization of prescribed drugs use among first generation elderly immigrants in the Netherlands

**DOI:** 10.1186/1472-6963-10-176

**Published:** 2010-06-22

**Authors:** Semiha Denktaş, Gerrit Koopmans, Erwin Birnie, Marleen Foets, Gouke Bonsel

**Affiliations:** 1 Department of Health Policy and Management, Erasmus University Rotterdam - Rotterdam, the Netherlands, PO Box 1738, 3000 DR Rotterdam, The Netherlands; 2 Department of Obstetrics & Gynecology, Erasmus MC - Rotterdam, the Netherlands, PO Box 2040, 3000 CA Rotterdam, The Netherlands

## Abstract

**Background:**

In developed countries, health care utilization among immigrant groups differs where the dominant interpretation is unjustified overutilization due to lack of acculturation. We investigated utilization of prescribed drugs in native Dutch and various groups of immigrant elderly.

**Methods:**

Cross-sectional study using data from the survey "Social Position, Health and Well-being of Elderly Immigrants" (the Netherlands, 2003). Ethnicity-matched interviewers conducted the survey among first generation immigrants aged 55 years and older. Outcome measure is self-reported use of prescribed drugs. Utilization is explained by need, and by enabling and predisposing factors, in particular acculturation; analysis is conducted by multiple logistic regression.

**Results:**

The study population consisted of immigrants from Turkey (n = 307), Morocco (n = 284), Surinam (n = 308) and the Netherlands Antilles (n = 300), and a native Dutch reference group (n = 304). Prevalence of diabetes mellitus (DM), COPD and musculoskeletal disorders was relatively high among immigrant elderly. Drug utilization in especially Turkish and Moroccan elderly with DM and COPD was relatively low. Drugs use for non-mental chronic diseases was explained by more chronic conditions (OR 2.64), higher age (OR 1.03), and modern attitudes on male-female roles (OR 0.74) and religiosity (OR 0.89). Ethnicity specific effects remained only among Turkish elderly (OR 0.42). Drugs use for mental health problems was explained by more chronic conditions (OR 1.43), better mental health (OR 0.95) and modern attitudes on family values (OR 0.59). Ethnicity specific effects remained only among Moroccan (OR 0.19) and Antillean elderly (OR 0.31). Explanation of underutilization of drugs among diseased with diabetes and musculoskeletal disorders are found in number of chronic diseases (OR 0.74 and OR 0.78) and regarding diabetes also in language proficiency (OR 0.66) and modern attitudes on male-female roles (OR 1.69).

**Conclusions:**

Need and predisposing factors (acculturation) are the strongest determinants for drugs utilization among elderly immigrants. Significant drugs underutilization exists among migrants with diabetes and musculoskeletal disorders.

## Background

Health care utilization in developed countries is known to differ between immigrant and indigenous groups [1]. In some cases, especially General Practitioner (GP) services, immigrant's utilization of health care is higher [2]; more often, however, utilization is lower than expected [3]. Lower utilization is commonly explained by more or higher thresholds immigrant groups may experience when seeking for medical help. Once medical help is provided, the nature or intensity of the care provided often varies by ethnic group, mediated by several medical and sociological processes.

Prescribed drugs are among the health services studied for the presence of such ethnicity-related utilization differences. High prescription variability has been reported among immigrant groups within Western countries including the Netherlands [4-7]. This variability cannot simply be explained by inequality of need alone. Whether drug utilization in general is increased among immigrants regardless the disease status, or whether specific patterns of over- and maybe underutilization exist, requires separating healthy and diseased persons in the analysis. Joining healthy and diseased persons into one analysis implicitly assumes that one common mechanism is responsible for utilization level in diseased persons and in healthy people. In fact a higher utilization level in diseased may point to adequate use, while it may point to overutilization in healthy. As most studies are population studies with overrepresentation of healthy subjects, these existing studies primarily explain overutilization in healthy persons.

This paper focuses on ethnicity related variation in the utilization of prescribed drugs, focussing on underutilization in diseased subjects as being different from overutilization in healthy persons. Furthermore we distinguished between mental and physical morbidity/drugs as acculturation-induced variation is present here. We used data from a group of first generation immigrants of 55 years and older, all from the four largest immigrant groups in the Netherlands: Turkish, Moroccan, Surinamese and Antillean immigrants. Our age selection had the advantage of sufficiently high prevalence of unambiguously diseased subjects among all ethnic groups. Our aim is to establish whether condition-specific utilization of defined drugs was lower among ethnic groups, to evaluate the relevance of several factors of utilization put forward, and to demonstrate whether so-called 'convergence' towards the utilization levels of the indigenous group is related to the degree of broadly measured acculturation. We used the well known Andersen's health care access model as analytic framework and hypothesized that health status would explain ethnicity-related drug utilization in terms of need, socio-economic status in terms of knowledge in general, while lack of acculturation could account for underutilization of care. We additionally explored whether ethnicity-related differences existed between the utilization patterns in case of mental vs. predominantly physical morbidity.

## Methods

### Conceptual model

**Figure 1:**
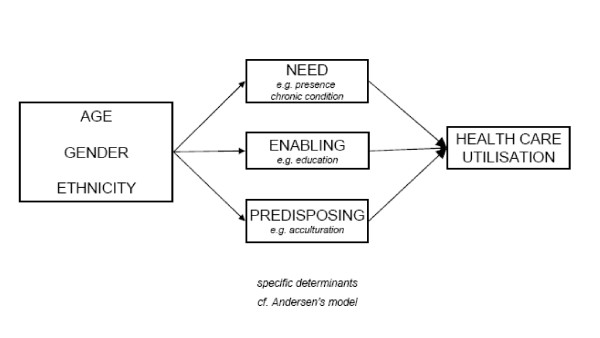
**Adapted Andersen model**.

Andersen's behavioural model [8] is presented in figure 1.

His model rests on three individual determinants of health care use, which we elaborate below illustrated by Dutch immigrant examples.

(1) *Need *refers to ill-health conditions or deficits in health status. Especially self-perceived health is relevant here, since it initiates the decision to seek care. Most elderly immigrants perceive their health worse then natives and they experience more limitations in Activities of Daily Living (ADL), pain chronic conditions and worse mental health [9,10].

(2) *Enabling factors *reflect the economic means (e.g., income) and human capital (e.g., education, knowledge) which enable people to utilize health services. In this context a lower socioeconomic position implies less knowledge on available services, less financial resources, and less self-reliance. In the Netherlands, first generation immigrants from Turkey and Morocco are low educated and women often are illiterate. Turkish and Moroccan elderly often have been unemployed for a long time and consequently have low income. Compared to Turkish and Moroccan elderly, Surinamese and Antillean elderly are better off resulting in an intermediate social economic position [11]. Under the Dutch health insurance system, the great majority of immigrants - in particular the elderly - fall under the public compulsory scheme, which fully covers prescribed drugs, without, at the time of data collection, copayment. Indigenous elderly are covered more often by a private insurance scheme, but last decade has shown that drug utilization is insensitive to the current low levels of copayment which are alleviated by tax compensation in case of chronic disease.

(3) *Predisposing factors*, the third determinant group, refers to the propensity of individuals to use services, including beliefs and attitudes regarding health and use of specific services. In the context of migrant's use of health services these attitudes primarily are a function of acculturation [12]. The general concept of acculturation, including acculturation in the domain of health care, is defined as "those phenomena which result when groups of individuals having different cultures come into continuous first-hand contact, with subsequent changes in the original cultural pattern of either or both groups" [13]. Since we focus on migrant use of health services, we added two complementary operationalisations of acculturation to the Andersen model, derived from Berry and Ester respectively. Berry [14] articulates the process of any migrant's acculturation into two decisions. The first pertains to the decision whether one maintains his or her own cultural identity. The second one involves the decision whether to engage in relations (contact and participation) within the larger society. Both decisions can co-exist, and strongly relate to (acquired) language proficiency. The gradual adaptation to modernity can be considered part of acculturation. 'Modernity' in Ester's [15] view is the most fundamental feature of Western societies and is defined as the transition of an agricultural to an (post)industrial society characterized by individualisation, secularisation, pluralisation, emancipation and democratisation [16,17]. Most of these processes also apply to health care. The dominant migrant groups in the Netherlands show different patterns of modernisation according to their background and generation [18,19].

### Data source and population

We used data from the "Social Position, Health and Well-being of Elderly Immigrants" survey, conducted in 2003 in the Netherlands[10,20]. To achieve a representative sample, we adopted a sampling method that has been used in large household surveys among immigrants in the Netherlands[21]. First, on the basis of municipality and region size, all municipalities in the Netherlands were classified into 16 strata with different percentages of immigrant persons. From these 16 strata, 9 strata with the highest percentage of the immigrants were selected. Secondly, within these 9 strata, for each migrant group separately, the 11 municipalities with the largest prevalence of that particular migrant group were selected. Ex post this strategy emerged into the same 11 municipalities, with, of course, slightly different patterns of ethnicity prevalences. Samples were drawn from the municipal population registers. Ethnic background was established by country of birth as documented in these registers. Compared to the Dutch population, immigrant elderly are less represented in the oldest age groups, while men are overrepresented because e.g. not all male immigrants were reunited with their spouses in the host country. Therefore, the sample was stratified into sex and two age groups (55-64 years and 65 years and older).

A total sample of 3284 people (808 Turks; 455 Moroccans; 688 Surinamese, 636 Antilleans and 697 Dutch) aged 55 years and above was drawn from the municipal registers. Of the 3284 subjects sampled, 1503 completed the questionnaire. The response rates were amongst Turkish 43.6%, Moroccans 65.3%, Surinamese 48.7%, Antilleans 54.2% and amongst native Dutch 47.3%. Excluding those with incorrect home addresses (amongst Turkish 5.6%, Moroccans 2.9%, Surinamese 3.9%, Antilleans 7.1% and Dutch 3.7%), the reasons for non-response were the following: (1) respondents could not be reached during the fieldwork: amongst Turkish 35.0%, Moroccans 16.2%, Surinamese 21.1%, Antilleans 22.7% and amongst Dutch 10.9%; (2) language problems: amongst Turkish 3.5%, Moroccans 0.7%, Surinamese 0.4%, amongst Antillean and Dutch 0%; (3) some elderly considered themselves too ill: amongst Turkish 6.7%, Moroccans 3.5%, Surinamese 8.4%, Antilleans 6.9% and amongst Dutch 8.6%; (4) respondents refused participation: amongst Turkish 11.3%, Moroccans 13.8%, Surinamese 21.4%, Antilleans 16.2% and amongst Dutch 33.1%; and finally other specified reasons: amongst Turkish 0.5%, Moroccans 0.4% and amongst Surinamese, Antilleans and Dutch 0%.

### Data collection method

The survey was translated into Turkish and Moroccan Arab and extensively tested in a pilot study. For the primary study 202 interviewers were trained: 61 native Dutch, 19 Antillean, 50 Moroccan, 27 Surinamese and 45 Turkish. Data were collected between April 2003 and December 2003. Trained interviewers from a similar ethnic background conducted structured face-to-face interviews at home. The respondents were approached personally on their home addresses for two reasons: (1) to enhance study participation, and (2) telephone possession and/or the amount of secret numbers among some ethnic groups are at a low respectively high level. Interviewers were instructed to pay visits during daytime and evening to avoid work-related non-response. If the respondent was absent, the interviewer was instructed to visit the same address on at least two further occasions. All respondents received a €5 gift certificate. Reluctance to participate was related to not being convinced of the usefulness, apparent oversampling of immigrant groups for other studies, and a changing societal context being clearly less tolerant towards immigrants.

### Measurements

The survey contained questions on prescribed pharmaceutical use, health status, socio-demographic background and acculturation, all self-report measures. The dependent variable was pharmaceutical use in the preceding 14 days (yes/no). Since we anticipated different patterns of utilization relevant factors particularly a larger role for acculturation in mental problems and mental drug use, we distinguished two types of pharmaceuticals: (1) pharmaceuticals prescribed in the case of physical chronic diseases [diuretics, heart drugs, skin drugs, rheumatoid arthritis drugs, allergy drugs, asthma drugs and insulin] and (2) pharmaceuticals prescribed in the case of mental health problems [psycho-pharmaceuticals and sleep medications]. Information on dosage was not included; in case of type 1 pharmaceuticals, we could compare head-to-head self-report presence of disease with the use of an indicated pharmaceutical (diabetes, COPD, musculoskeletal disease).

The independent variables were measured as follows. Three indicators of health status were included: self-rated health as measured by a single-item question 'In general would you describe your health as: excellent, very good, good, poor, very poor [22]. Secondly the number of self-reported chronic conditions from which the respondents suffered in the 12 months preceding the interview (ranging from 0 to 11) [23]. Finally, mental health was measured by the SF-12 Mental Component Summary (MCS) which is composed of four questions referring to the past 4 weeks: (1) Have you felt calm and peaceful? (All of the time, most of the time, a good bit of the time, some of the time, a little of the time, or none of the time); (2) Did you have a lot of energy?; (3) Have you felt downhearted and blue?; (4) How much of the time has your physical health or emotional problems interfered with your social activities like visiting with friends, relatives etc? A higher MCS (range: 0-100) indicates better mental health [22].

The second group of independent variables consisted of indicators of socio-economic position: educational level and monthly household income [24]. Educational level concerned the highest degree achieved (no education/primary education, lower secondary education, higher secondary education, and higher vocational college/university). Household net income was standardized for the number of persons in the household.

The third group consisted of our two acculturation concepts, operationalized into 5 domains and measured accordingly through validated questions[11,19]: (1) mastery of Dutch language as a proxy for contact with native Dutch, (2) religiosity, (3) attitudes on care for family, (4) attitudes on male-female role and (5) attitudes on family values. Dutch language proficiency was measured among Turkish and Moroccan elderly by three questions: (1) when someone talks to you in Dutch, are you able to understand (yes often, yes sometimes, no); (2) do you have difficulty in speaking Dutch (yes often, yes sometimes, no); (3) when you read a Dutch paper or a letter do you have difficulty in understanding (yes often, yes sometimes, no). A summated score was calculated which was subsequently recoded in 3 categories indicating the relative level of mastery of Dutch language (1) poor, (2) mediocre, (3) good. Since most Surinamese and Antillean elderly speak fluently Dutch because of their colonial background, we used the following proxy question to evaluate Dutch language proficiency among Dutch, Surinam and Antillean elderly: Did you fully understand the GP (yes/no) during the last GP visit? If no, the proficiency variable was coded 'mediocre', otherwise 'good'.

Religiosity was measured by asking whether one considers oneself as belonging to a religion (yes/no). Attitudes regarding care for family, male-female-roles and family values were measured by means of a set of 14 statements, e.g., children should take care of their parents when they are old, an education is more important for boys than for girls (with a Likert type response mode: agree, partly agree/partly disagree, do not agree). A summated score was calculated and subsequently recoded in 3 categories indicating a (1) traditional, (2) moderate traditional, (3) modern attitudes on the above values.

### Analysis

First we described the socio-demographic status, acculturation, and self-perceived health according to ethnic background of the full sample. Additionally, we described ethnicity-related under-utilization in three specific chronic conditions: diabetes mellitus, Chronic Obstructive Pulmonary Disease (COPD), and musculoskeletal disorders. As disease-specific pharmaceutical treatment is generally mandatory in all three diseases, we defined underutilization as the lack of specific treatment. Next, the aim of the first general analysis was to explain drug utilization for non-mental chronic conditions and mental conditions separately. The roles of need (presence of any chronic condition), enabling (higher socio-economic class, higher education) and predisposing factors (here: indicators of acculturation) were determined, and compared between drugs utilization for mental and chronic disease separately. The second, explanatory analysis investigated these associations among respondents with any of the three previously mentioned specific conditions, to detect drug underutilization.

All explanatory analysis applied conventional multiple linear logistic regression models (method enter), with presence/absence of drug utilization as the dependent variable. The crude and adjusted odds ratios (95% Confidence Intervals) are the primary measure to express the strength of the association. The analyses were performed using SPSS 13.0 for Windows. A two sided p-value < 0.05 was considered a statistically significant difference.

## Results

The study included 304 native Dutch, 307 Turkish, 284 Moroccan, 308 Surinamese and 300 Antillean elderly (see table 1).

**Table 1:** Socio-demographic characteristics and socio-economic status, acculturation and, self-perceived health by ethnic background in the Netherlands (2003)

	NETH (n = 304)	TURK (n = 307)	MOROC (n = 284)	SURI (n = 308)	ANTIL (n = 300)	*p-value
**Socio-demographics**						
Age (55-64y) (%)						0.904
Men	47.1	51.3	43.8	45.0	48.6	
Women	47.3	49.3	51.8	50.5	51.9	
**Socio-economic status**						
No education (%)	17.3	70.5	94.0	37.2	39.0	< 0.001
Primary education (%)	14.0	12.5	3.2	11.9	9.9	
Lower secondary education (%)	33.0	14.9	0.7	21.8	19.9	
Higher secondary education (%)	20.3	1.0	1.8	17.2	17.0	
Higher vocational college/university (%)	15.3	1.0	0.4	11.9	14.2	
Standardised net income per month in €, mean (sd)	1226 (497)	708 (215)	571.(193)	952 (425)	967 (500)	< 0.001
**Acculturation**						
Mastery of Dutch language (%)						< 0.001
Poor	0.0	48.2	44.7	0.0	0.0	
Mediocre	1.3	48.5	49.6	2.4	1.6	
Good	98.7	3.3	5.6	97.6	98.4	
Religious (%)	47.2	97.7	99.6	90.5	88.3	< 0.001
Attitudes on care for family (%)						< 0.001
Traditional	3.6	40.7	55.9	12.1	21.5	
Moderate traditional	36.3	48.3	41.6	57.0	47.0	
Modern	60.1	10.9	2.5	30.9	31.5	
Attitudes on male-female roles (%)						< 0.001
Traditional	13.9	47.4	45.4	13.7	8.1	
Moderate traditional	29.7	35.1	25.7	35.9	36.2	
Modern	56.4	17.5	28.9	50.3	55.7	
Attitudes on family values (%)						
Traditional	11.3	30.7	36.2	20.9	14.3	< 0.001
Moderate traditional	61.6	58.1	63.1	65.9	70.4	
Modern	27.2	11.2	0.7	13.2	15.3	
						
**Health status**						
Self-rated chronic conditions, mean (sd)	1.7(1.6)	3.4 (2.0)	2.8 (1.8)	2.3 (1.8)	1.6 (1.4)	< 0.001
MCS SF-12, mean (sd)	51.7 (11.4)	41.6 (11.6)	42.0 (10.0)	46.2 (12.9)	49.7 (11.1)	< 0.001

*χ^2 ^test was performed

Immigrant elderly, particularly from Turkish or Moroccan descent, had a lower socio-economic position. The degree of acculturation also differed according to ethnic group. Parallel to socio-economic inequalities, large differences in Dutch language proficiency exist. While Surinamese, Antilleans were relatively good Dutch speaking as expected, the mastery of Dutch among Turks and Moroccans was mediocre to low. Compared to native Dutch all immigrant elderly groups were more religious. Moreover, immigrant elderly, especially Turks and Moroccans, more often reported traditional attitudes on care for family, male-female roles and family values. Inequality of health was abundant; Turkish, Moroccan and Surinamese elderly more often reported poor health and more chronic conditions than native Dutch. Especially Turkish and Moroccan elderly reported relatively poor mental health. Indigenous and Antilleans showed the highest prevalence of a healthy state.

**Figure 2:**
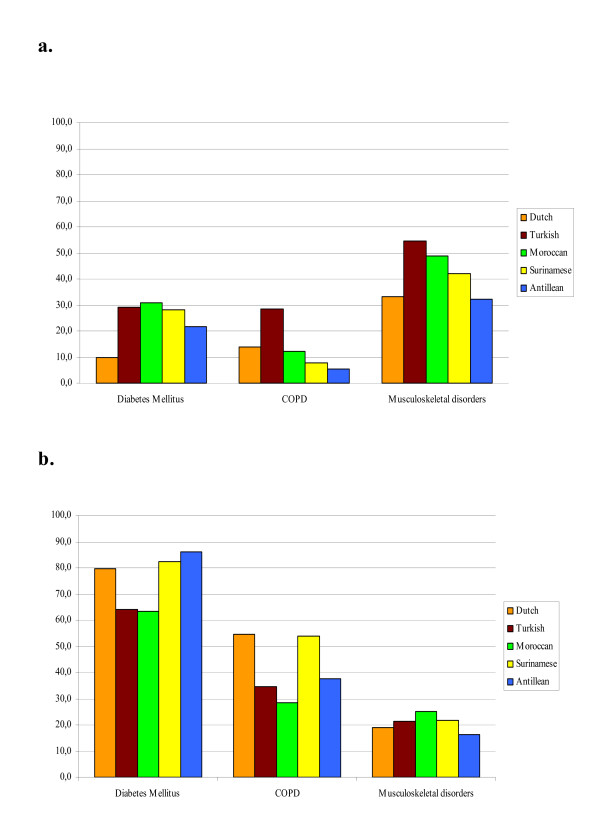
**Panel a: Prevalence of three self-reported chronic conditions and panel b: specific drug utilisation for that condition among the diseased (panel a) five ethnic groups (Dutch N = 49; Turkish N = 168; Moroccan N = 132; Surinamese N = 145; Antillean N = 93) in the Netherlands (2003)**.

Figure 2a illustrates the higher prevalence of diabetes mellitus in all immigrant elderly groups. As Figure 2b depicts, drugs utilization among diseased (the prevalent cases of Figure 2a) was lower in Turkish and Moroccan elderly as compared to the other immigrant groups. Prevalence of COPD was high among Turkish elderly, but again the related drugs utilization of Turkish was relatively low. Finally, the prevalence of musculoskeletal disorders was higher among Surinamese, Moroccan and Turkish elderly. Unlike diabetes and COPD, drug utilization for musculoskeletal disorders was comparatively higher in most of the immigrant groups with this condition. All differences were statistically significant (p < 0.001).

Table 2 shows the impact of need, enabling and predisposing factors and the additional impact of specific ethnic background (full model) on drugs utilization for chronic diseases. We first discuss the results without adjusting for ethnic background (not shown in table). Drugs utilization was significantly associated with more self-rated chronic conditions (OR 2.55), higher age (OR 1.03), and better Dutch language proficiency (OR 1.63). Modern attitudes on male-female roles (OR 0.76) and religiosity (OR 0.87) were significantly associated with lower utilization of drugs for chronic diseases. When adjusted for specific ethnic background (full model), more chronic conditions (OR 2.64), higher age (OR 1.03), modern attitudes on male-female roles (OR 0.74) and religiosity (OR 0.89) significantly contributed to drug utilization. Only Turkish background (OR 0.42) appeared to play an additional, ethnic-specific role, lowering utilization. Testing for interaction between need factors and ethnic group resulted in two statistically significant interactions: between Turkish background and number of chronic diseases (OR 0.44; CI 0.27-0.71; p < 0.01) and between Moroccan background and number of chronic disease (OR 0.56; CI 0.34-0.91); p < 0.05). The explanatory analysis of drugs use for mental health problems showed an almost identical pattern, apart from the more pronounced specific ethnic effects in persons from Moroccan (OR 0.19) and Antillean (OR 0.31) descent. Interaction between need factors and ethnic background was also tested. Only one interaction appeared significant: between Moroccan background and self rated mental health (OR 0.95; CI 0.91-0.99; p < 0.05).

**Table 2:** Prescribed drug utilization for chronic diseases and for mental health problems, assessed by multiple logistic regression (N = 691; Odds Ratios and 95% Confidence Intervals).

	Drugs use for chronic diseases	Drugs use for mental health problems
**Need factors & basic demographics**	**Full model**	**Full model**
No.self-rated chronic conditions (cf. prescribed list, range 0-11)	2.64 (2.31-3.02)***	1.43 (1.18-1.74) ***
Self-rated mental health (MCS SF12, range: 0-100; a higher score represents better mental health)	1.00 (0.99-1.02)	0.95 (0.93-0.97)***
Male	1.13 (0.84-1.52)	1.17 (0.71-1.83)
Age (years)	1.03 (1.01-1.05)**	1.00 (0.98-1.03)
**Enabling factors**		
Educational level (no/primary education vs secondary and higher education)	0.93 (0.65-1.34)	0.99 (0.62-1.97)
Standardized net household income (Euros)	1.00 (1.00-1.01)	1.00 (0.99-1.01)
**Predisposing factors**		
Good Dutch language proficiency	0.97 (0.66-1.43)	1.64 (1.19-2.58)
Modern attitudes on care for family	0.98 (0.77-1.24)	1.04 (0.82-1.73)
Modern attitudes on male-female roles	0.74 (0.60-0.91)**	1.01 (0.65-1.30)
Modern attitudes on family values	0.84 (0.64-1.11)	0.59 (0.40-0.95)*
Religiosity	0.89 (0.80-0.97)**	0.93 (0.79-1.10)
**Ethnic background **(Dutch = reference)		
Turkish	0.42 (0.19-0.95)*	0.50 (0.13-1.99)
Moroccan	0.54 (0.24-1.21)	0.19 (0.04-0.81)*
Antillean	1.59 (0.99-2.57)	0.31 (0.13-0.76)*
Surinamese	1.44 (0.88-2.34)	0.54 (0.26-1.12)

*p < 0.05, **p < 0.01, ***p < 0.001

The explanatory analysis of drug underutilization in three specific conditions is showed in Table 3. In diabetes mellitus, a higher number of self-rated chronic conditions (OR 0.74) and good Dutch language proficiency (0.66) were associated with lower underutilization, whereas modern attitudes on male-female roles (OR 1.69) was associated with higher underutilization of drugs for DM. In COPD none of the proposed variables explained the presence of drugs underutilization. In musculoskeletal conditions more self-rated chronic conditions (OR 0.78) significantly contributed to lower underutilization of drugs for musculoskeletal disorders.

**Table 3:** Prescribed drug underutilization for diabetes mellitus, COPD and musculoskeletal disorders, assessed by multiple logistic regression (Odds Ratios and 95% Confidence Intervals).

	Underutilization of drugs for DM (N = 274)	Underutilization of drugs for COPD (N = 161)	Underutilization of drugs for musculo skeletal disorders (N = 483)
**Need factors & basic demographics**			
No. of self-rated chronic conditions (cf. prespecified list; range 0 - 11)	0.74 (0.56-0.98)*	1.03 (0.76-1.40)	0.78 (0.64-0.93)**
Self-rated mental health (range: 0 to 100; a higher score represents better mental health)	0.98 (0.96-1.01)	0.99 (0.97-1.03)	1.01 (0.99-1.03)
Male	1.19 (0.63-2.24)	1.03 (0.49-2.14)	1.40 (0.87-2.25)
Age (years)	0.99 (0.96-1.02)	1.03 (0.99-1.07)	1.00 (0.97-1.02)
**Enabling factors**			
Educational level (no/primary education vs secondary and higher education)	0.53 (0.22-1.24)	0.68 (0.27-1.72)	1.04 (0.45-1.59)
Standardized net household income (Euros)	1.00 (0.99-1.01)	1.00 (0.99-1.00)	1.00 (0.99-1.01)
**Predisposing factors**			
Good Dutch language proficiency	0.66 (0.43-0.996)*	0.85 (0.50-1.43)	0.88 (0.62-1.23)
Modern attitudes on care for family	0.71 (0.43-1.16)	0.72 (0.42-1.23)	0.92 (0.64-1.33)
Modern attitudes on male-female roles	1.69 (1.08-2.66)*	1.07 (0.66-1.76)	1.32 (0.94-1.84)
Modern attitudes on family values	1.57 (0.89-2.75)	1.05 (0.58-1.89)	0.96 (0.64-1.46)
Religiosity	1.04 (0.86-1.30)	1.05 (0.83-1.34)	0.93 (0.80-1.08)

*p < 0.05, **p < 0.01, ***p < 0.001

## Discussion

This study on drugs use among the four major elderly immigrant groups in the Netherlands shows considerable ethnicity-related variation in prescribed drug utilization.

Within three specific chronic disease categories, we found evidence of underutilization among immigrant groups.

The augmented Andersen model proved useful in explaining these general and disease-specific patterns. Foremost, the prevalence of chronic diseases for which drug treatment is available is generally higher in ethnic groups and this health status factor (*need *factor) primarily explains ethnicity related variation. So-called *enabling *factors, in particular education and income, do not add to the explanation of drugs use. Acculturation as *predisposing *factor, however, was effective in explaining intergroup variation.

Three components of acculturation contributed to drug use: good language proficiency, modern attitudes on male female roles and religiosity. From the results of specific diseases it could be deduced that language proficiency primarily reduced the observed underutilization of among ethnic groups. Unexpectedly, modern attitudes on male-female roles enhanced underutilization.

Apparently, being able to communicate properly with the doctor enhances the likelihood of patients to get drug therapy. The consistent utilization-lowering effect of modern attitudes regarding male-female roles is difficult to interpret. We can offer one potential explanation: this attitude question selects a specific group of higher-educated elderly with modern attitudes, which - more than we could account for by the standard education question - decrease drug use (residual confounding).

In a local study with Reijneveld [1] reported a similar strong effect of need to explain drug utilization in an Amsterdam sample of elderly immigrants. Our study adds the significant contribution of acculturation, especially language proficiency, to drugs use. Comparison with other continental studies is limited since this is the first European study focusing on disease specific drugs use in an ethnic diverse elderly population. However, the lower rate of drugs use among immigrant elderly without command of the native language is consistent with results from similar studies in the US among non-native language speaking immigrant groups [25-28]. A study conducted in Turkey among elderly diabetics also found underutilization of drugs indicating a trend among physicians to under prescribe insulin [29]. Since our study did not address prescribing behaviour of physicians we do not know if underutilization among immigrant elderly in the Netherland can be explained by lack of knowledge among physicians.

There are several limitations to our study. First we used self-reported survey data on both the prevalence of chronic diseases and the drug utilization, without clinical data or pharmaceutical registries to verify accuracy. Kriegsman et al. (1999)[30], however, reported adequate accuracy and validity of patients' self-reports on the presence of specific chronic diseases, using essentially the same questions. Respondent's report of a musculoskeletal disorders was confirmed by GP standards, according to Hughes et al. (1993)[31]. Reijneveld (2000) [32] and Wagner [33] also showed self-report use of prescription drugs to be fairly accurate in general, and among ethnic minority groups. Only in case of the mental domain, some underestimation of disease and treatment could result from ethnicity-related reluctance to report. We therefore do not expect our results to be biased due to the general reliance on self-report data.

Secondly, a related disadvantage of asking the presence/absence of a condition is the lack of information on the severity or the disease stage of the reported chronic disease, both which usually affect the likelihood of drugs treatment. Our examples, however, represent diseases for which drug treatment is standard practice.

As a third issue one could challenge, is our deliberate use of registered country of birth as indicator of immigrant background. As opposed to self-assessment the major advantages are high reliability and lack of missing information. It validly assumes culturally homogeneous groups in case of Moroccans and Turks but neglects ethnic subgroups within the Surinamese group (South-Asian Hindustani, African Creoles). However, in our context they share an important acculturation factor: adequate language proficiency, hence the effect of the disadvantage may be small in our context. First vs. second generation issues were not relevant in this study, as the share of second generation immigrants in elderly population is small. We admit that the relevance of this comparison over time will increase.

A fourth limitation is the non-response rates. The age/sex distributions of our samples were as expected due to the sampling procedure, indicating absence of selective non-response. The most frequent reason for non-response was the respondent's absence at the address at the time of visit and less frequent being ill and refusal. While non-response is likely to lower disease prevalence in the responding group, we think it is unlikely that the primary relation between explanatory factors and drugs use is different for diseased respondents vs. non-respondents. Two previous studies in the context of population-based research among immigrants demonstrated small to ignorable effects of selective non-response on these types of outcome variables [34,35].

Finally, by focussing on the diseased, we ignored those without self reported disease, yet using drugs. These cases of our sample are interesting enough, but additional information is mandatory to safely analyze and interpret these cases. Such respondents could suffer from some disease not included on our list or misinterpret our question on chronic disease -in that case utilization is valid. It is also possible that these respondents are truly without disease -in our terms- and their drug use remains to be explained. Qualitative research is indicated in our view.

Overall, our results fitted to our hypothesis, but some findings were unexpected.

First is the overriding impact of reported health status (need) compared to e.g. socio-economic factors (enabling). We regard it reassuring as it primarily implies that health care inequalities in our study reflect health status inequalities.

Second, the augmentation of the Andersen model with various acculturation factors had one unexpected result. Acculturation indeed was important with language proficiency as a tool to access. Surprisingly, modern attitudes appeared to have effects opposed to our expectation (see above). The 'simple' education variable did not show relevant effects which may be the consequence of introducing the language variable. If true, in that case the education pathway is different between immigrants and the indigenous group.

On the practical level, the pattern of systematic and sizable underutilization is a challenge for health care providers and policy makers. Non-Dutch speaking first generation immigrant patients should be recognized as a high-risk group for inadequate care, even if they stay a lifetime in the country. Generally, intervention targets are present at both sides: newcomers should be offered facilities to learn and improve language skills, while long stay first generation immigrants most likely have to rely on peer educators and translation services.

## Conclusions

This study is among the first to investigate at the national level, differences in drugs use between immigrant and native elderly. The bad news is that immigrants are in a disadvantaged position regarding disease prevalence and drugs use and predominantly will remain so if language proficiency is insufficient, even if being resident for a long time in the Netherlands. The good news is that we believe this disadvantage can be rationally addressed.

## Competing interests

The authors declare that they have no competing interests.

## Authors' contributions

SD and MF participated in the design of the study, SD performed the statistical analysis with GK and SD drafted the manuscript. EB and MF commented on the draft. GB assisted in drafting the manuscript. All authors read and approved the final manuscript.

## Pre-publication history

The pre-publication history for this paper can be accessed here:

http://www.biomedcentral.com/1472-6963/10/176/prepub
